# Associations Between Big-5 Personality Traits, Cognitive Ability, and Climate Beliefs and Behaviours: Results From a Longitudinal UK Birth Cohort

**DOI:** 10.5964/ejop.13657

**Published:** 2024-11-29

**Authors:** Ken Freminot, Katie Major-Smith, Kate Northstone, Isaac Halstead, Daniel Major-Smith

**Affiliations:** 1 Population Health Sciences, Bristol Medical School, University of Bristol, Bristol, United Kingdom; 2 Sustainability, Creativity and Innovation Research Group, Plymouth Marjon University, Plymouth, United Kingdom; 3 Centre for Academic Child Health, Population Health Sciences, Canynge Hall, Bristol Medical School, University of Bristol, Bristol, United Kingdom; University of Johannesburg, Johannesburg, South Africa

**Keywords:** ALSPAC, individual differences, personality, cognitive ability, climate change, sustainability

## Abstract

Anthropogenic climate change is an existential threat to both humans and wider biodiversity. However, cumulatively, individuals’ actions can help to mitigate the impacts of climate change. Understanding the factors which shape individuals’ beliefs about climate change, and their environmental behaviours, is therefore crucial. Here, we explore whether individual differences—Big-5 personality traits and cognitive ability—are associated with climate beliefs and behaviours, using longitudinal data from a UK birth cohort study. Individual differences were measured when the participants were teenagers (aged 13 to 15 years), with climate beliefs and behaviours assessed at approximately age 30 years. These climate beliefs and behaviours included: belief that the climate is changing, concern over climate change, whether humans are to blame for climate change, whether individual actions can mitigate climate change, and whether they were undertaking a range of pro-environmental behaviours for climate reasons (e.g., reducing air travel, reducing meat/dairy consumption). Regression models were used to explore the associations between individual differences and climate belief and behaviour outcomes, adjusting for a range of relevant sociodemographic confounders. Overall, we found consistent positive associations between agreeableness, openness to experience and cognitive ability scores and environmental knowledge and action. Weaker, and more inconsistent, associations were reported for extraversion, conscientiousness and emotional stability. These results suggest that individual differences may shape an individual’s beliefs and actions regarding climate change, and potentially indicates groups where climate information campaigns could be targeted.

Climate change represents both an ecological and existential threat to human and non-human animals. Driven by human activity, the impacts of climate change are intensifying, causing an increasing array of environmental, social and economic issues worldwide, including loss of livelihood, negative health impacts and potentially irreversible environmental damage (IPCC, 2023). While the Paris Agreement aims to address this crisis by mobilising international action (UNFCCC, 2016), and governments have adopted net-zero commitments worldwide to meet this agreement (UNEP, 2023), atmospheric concentrations of greenhouse gas emissions are at their highest levels compared to the previous million years (IPCC, 2023), emphasising the urgent need to take stronger action to mitigate the climate crisis.

An important aspect to addressing climate change is individual action (Hampton & Whitmarsh, 2023). Sustainable action among the public can collectively have a large impact on reducing climate change (Boehm et al., 2023), with actions such as eating a fully plant-based diet, driving less and reducing plane travel, significantly reducing an individual's carbon footprint (Ivanova et al., 2020; Wynes & Nicholas, 2017). While macro-level systemic changes are needed to stop anthropogenic climate change (such as industry transformation and stronger government regulations; Chater & Loewenstein, 2023), individual action is a core component to helping achieve global net-zero targets, with the Intergovernmental Panel on Climate Change (IPCC) advocating a shift among the public to more sustainable behaviour and less emission intensive consumption (IPCC, 2023). Since individual action is important for helping mitigate climate change, understanding the characteristics that may predispose individuals to support or oppose climate change policies and engage with pro-environmental behaviours to minimise their personal impact on climate change is needed.

Several studies have been conducted to identify factors that predict pro-environmental attitudes and behaviours. For example, several demographic variables such as age, gender, and education have been found to be associated with environmental attitudes, with women, younger individuals, and those with greater educational attainment displaying greater concern regarding climate change (Poortinga et al., 2019). Researchers have also investigated the role of individual difference variables, such as personality. Personality traits are often measured using the Big-5 personality trait model, which is comprised of 5 personality traits formed from many facets of personality. These personality traits can be briefly summarised as Openness (being open to new ideas and experiences), Conscientiousness (a preference for order, attention to detail, and self-discipline), Extraversion (gregarious, outgoing, optimistic), Agreeableness (trusting, compliant, altruistic), and Neuroticism (impulsive, self-conscious, and pessimistic; note that some authors refer to this trait as ‘Emotional Stability’, which is the inverse of ‘Neuroticism’). When examined in the context of pro-environmental attitudes and behaviours, the most commonly associated traits are openness, agreeableness, and conscientiousness, all of which have been found to be positively associated with climate beliefs and actions; however, there is a high degree of heterogeneity in study findings (Soutter et al., 2020).

Some of this inter-study variability may be accounted for by limitations found in the existing literature. For example, many studies in this area are based upon small sample sizes (e.g., less than 500 participants; Soutter et al., 2020), which may prevent smaller effect sizes from being reliably detected (e.g., *r* = .15, which are typical in both personality research in general (Gignac & Szodorai, 2016) and specifically in environmental attitudes research Soutter et al., 2020). Many studies also focus on either pro-environmental attitudes or behaviours separately. This can be an issue when accounting for the large disconnect between attitudes and behaviours, especially in environmental research, where holding a positive attitude towards pro-environmental behaviours would not make a meaningful impact compared to actually performing those behaviours (Whitmarsh et al., 2021). Additionally, previous environmental attitudes and behaviour research is largely cross-sectional in nature, assuming the associations between personality and environmental attitudes to be unidirectional (i.e., personality causes environmental attitudes and behaviours), and often with little adjustment for potential confounders (Soutter et al., 2020). Longitudinal studies that measure personality (or any other exposure; the term ‘exposure’ is largely synonymous with ‘independent variable’) *prior* to measuring environmental beliefs and behaviours may be able to provide more evidence for a potential causal relationship between individual difference variables and environmental attitudes or behaviour.

In addition, previous studies have also neglected the role of intelligence (or ‘cognitive ability’) in pro-environmental attitudes and behaviours. Intelligence can be considered as a measure of an individual’s ability to acquire knowledge and make decisions (Deary et al., 2009). In the context of environmental attitudes and behaviours, cognitive ability may aid an individual in acquiring accurate information regarding climate change, its impact, and ways to mitigate it, as well as the actions that can be performed by an individual for this to be achieved. Furthermore, as openness is one of the most consistent and strongly associated traits with environmental attitudes and behaviours, and openness is also moderately related to intelligence (Furnham & Cheng, 2016), it is conceivable that intelligence may be another candidate variable for predicting environmental attitudes and behaviours.

The present study seeks to address the limitations of previous work by using a large, longitudinal cohort study that measured individual difference variables in childhood and environmental attitudes and behaviours in adulthood. It will also expand upon previous work that has investigated the role of openness, by also exploring the role of intelligence.

## Method

### Participants

Pregnant women resident in the former county of Avon (southwest UK) with expected dates of delivery between 1st April 1991 to 31st December 1992 were invited to take part in the ALSPAC study (the Avon Longitudinal Study of Parents and Children). The initial number of pregnancies enrolled was 14,541, of which there were a total of 14,676 foetuses, resulting in 14,062 live births and 13,988 children who were alive at 1 year of age (Boyd et al., 2013; Fraser et al., 2013). When the oldest children were approximately 7 years of age, an attempt was made to bolster the initial sample with eligible cases who had failed to join the study originally, resulting in an additional 913 children being enrolled. The total sample size for analyses using any data collected after the age of seven is therefore 15,447 pregnancies, resulting in 15,658 foetuses, of which 14,901 were alive at 1 year of age (Northstone et al., 2019). These offspring are the focus of the present study.

Participants were excluded if they were not alive at 1 year of age, were from triplet or quadruplet pregnancies (for confidentiality reasons), or if they or their mother had withdrawn consent for their data to be used; this resulted in a final sample size of 14,834 ALSPAC offspring. Of this full sample, only participants with complete exposure, outcome and confounder data were included in the analytic sample (*n* = approx. 2,100 to 2,400, depending on the exposure and outcome combination). This reduction in sample size is due to a number of reasons, including: i) participants opting-out or losing contact with the study, and hence not being invited to participate in ALSPAC data collections; ii) participants being invited to take part in data collections, but declining to participate; iii) participants taking part in data collections, but not answering the relevant questions or sections; and iv) many participants also take part in some data collections but not others (e.g., they may have climate change beliefs/behaviours data, but no data on personality).

Please note that the study website contains details of all the data that is available through a fully searchable data dictionary and variable search tool at ALSPAC (2024). Study data gathered since the study offspring were aged 22 were collected and managed using REDCap electronic data capture tools hosted at the University of Bristol (Harris et al., 2009).

### Personality and Cognitive Ability Exposures

Personality was measured using the 50-item International Personality Item Pool (IPIP) questionnaire to assess the ‘Big-5’ personality traits (Goldberg, 1992). Data were collected via self-report on a computer at an ALSPAC study clinic when the study children were approximately 13 years of age. Of all ALSPAC offspring, ~11,300 (~75%) were invited to this clinic, of which ~6,100 (~55%; ~40% of the total sample) attended. The majority of those who attended (> 90%) had complete personality data. Response options were on a five-point Likert scale from ‘very like me’ to ‘not like me at all’ (for all items and response options, see Table S1, found in the Supplementary Materials section; Freminot et al., 2024). Each personality trait consisted of 10 items, which were summed to give the total score for said personality trait (i.e., between 10 and 50). Internal consistency assessed via Cronbach’s alpha was either ‘acceptable’ or ‘good’ (Agreeableness = 0.73; Conscientiousness = 0.75; Openness = 0.75; Emotional Stability = 0.83; Extraversion = 0.85).

Cognitive ability was assessed by the Wechsler Abbreviated Scale of Intelligence (Wechsler, 1999) at a clinic when the children were approximately 15 years of age. Of all ALSPAC offspring, ~10,700 (~70%) were invited to this clinic, of which ~5,500 (~50%; ~35% of the total sample) attended. The majority of those who attended (> 95%) had complete cognitive ability data. This measure consisted of two subtests to measure the child’s ‘intelligence quotient’ (IQ). These tests were conducted face-to-face by a trained fieldworker, and included the following tasks: a vocabulary task (understanding the meaning of a range of words) and a matrix reasoning task (i.e., pattern recognition). Scores on these tests were then standardised by age and converted to IQ scores with approximately a mean of 100 and standard deviation of 15.

All exposures were analysed as continuous variables (i.e., scores for each of the 5 personality traits and IQ scores for cognitive ability).

### Climate Change Beliefs and Behaviours Outcomes

Questions on climate beliefs and behaviours were collected via questionnaire between November 2021 and May 2022 when the participants were approximately 30 years of age. Of all ALSPAC offspring, ~9,000 (~55%) were invited to complete this questionnaire, of which ~4,300 (~50%; ~30% of the total sample) returned a questionnaire. The majority of those who returned a questionnaire (> 98%) had completed at least some of the questions in the section on climate change beliefs and behaviours. These questions consisted of a range of topics regarding beliefs on climate change and its causes and impact, including:

- Do you believe that the climate is changing? (responses: *Definitely not* vs. *Yes maybe* vs. *Yes probably* vs. *Yes definitely*).
- How concerned are you about the impact of climate change? (responses: *Not at all concerned* vs. *Not very concerned* vs. *Somewhat concerned* vs. *Very concerned*).
- Do you believe that humans are to blame for climate change? (responses: *Not at all* vs. *Yes, for some of it* vs. *Yes, for most of it* vs. *Yes, for all of it*).
- Do you think that what you do, however small, will make a difference to the long-term effects of changes to our climate? (responses: *No* vs. *Not sure* vs. *Yes*).

Participants were also asked whether they undertook a range of pro-environmental behaviours (e.g., travel, recycling, diet) for climate and/or non-climate reasons. All climate questions used in the present study are summarised in Table S2. Many of these questions were adapted from Bristol City Council’s Quality of Life Survey 2019 (BCC, 2020), with all others—Questions 1, 2b, 2c and 4g to 4q (Table S2)—developed in-house by the ALSPAC team. For more information on these climate change belief and behaviour questions, see (Major-Smith, Halstead, et al., 2024).

The climate belief questions were analysed on their original scale. For the climate behaviour questions, we categorised and analysed them in three ways: i) exploring each action as a four-level categorical variable (Not done action vs. Action taken due to climate change vs. Action taken for other reasons vs. Action taken due to climate change and for other reasons); ii) exploring each action as a binary variable (whether action taken due to climate change or not); and iii) as a ‘total number of actions taken for climate reasons’ score, by summing together the number of actions taken for climate reasons (excluding ‘other reasons’, as fewer participants answered this question).

### Confounders

While the aim of this paper is primarily descriptive, we will adjust for a range of key sociodemographic variables to try and remove some common sources of plausible confounding, which may provide stronger evidence for a causal interpretation compared to studies which do not adjust for confounders. These include the participants’ sex assigned at birth and their ethnicity, in addition to their mother’s age at birth of the child, home ownership status, highest educational attainment, and area-level index of multiple deprivation quintiles during pregnancy (see Table S3 for coding details). All confounders were measured during pregnancy, either abstracted from medical records (offspring sex), based on postcode information (deprivation) or self-reported questionnaires (all other confounders). As these confounders were measured early in the study, few participants have missing data for these variables (< 20% missing). While not a confounder, as it may impact climate beliefs and behaviours, we also adjusted for the participant’s age at completion of the questionnaire including climate questions to try and improve precision in our estimates.

### Analysis

Our main analyses were a series of regression models specific to the outcome of interest (e.g., ordinal models for ordered categorical outcomes, multinomial models for unordered categorical outcomes, and logistic regression for binary outcomes). Models were repeated both unadjusted and adjusted for all potential confounders. We used pseudo-R2 values to estimate the improvement in model fit when including the exposure of interest. For ordinal models, we tested the proportional odds assumption via the Brant test; if it was found to be violated (i.e., *p* < .05), analyses were repeated using multinomial models. For multinomial models, we used a likelihood ratio test to assess whether there was an overall association between the exposure and outcome. As the coefficients of ordinal and multinomial models are not necessarily intuitive to interpret, we converted these models to predicted probabilities of each outcome category to improve interpretability.

For the ‘total number of actions taken for climate reasons’ score, we initially modelled this using a linear regression model. However, as the distribution was non-normal (i.e., an excess of zeros; see ‘Results’ section) we explored a range of sensitivity analyses to see how robust these results were to different modelling specifications. First, as the data are technically counts, we used both Poisson and negative binomial models (the latter to account for potential over-dispersion). However, this model may also be mis-specified due to the excess zeros; we therefore also performed a zero-inflated Poisson and zero-inflated negative binomial regressions to model both the count data and the excess zeros. Despite the risk of potential model misspecification, as all models produced largely equivalent results we focus primarily on the linear regression results in the main text for ease of interpretation, with the count model results presented in the Supplementary Information (Freminot et al. 2024).

Rather than arbitrarily dichotomising our observational results into ‘significant’ and ‘non-significant’ (e.g., based on a *p*-value < .05, for instance), throughout this paper we interpret *p*-values as a continuous measure of the strength of evidence against—or incompatibility with—the null hypothesis of no association between the exposure and outcome (Sterne & Davey Smith, 2001). The only exception was for the Brant test, where we used *p* < .05 as a cut-off to indicate potential violation of the proportional odds assumption. Analyses were conducted in Stata Version 18, other than the creation of the synthetic datasets which was performed using R 3.4.1 (R Development Core Team, 2021).

## Results

### Descriptive Statistics

A summary of the sociodemographic profile of participants can be found in Table S3. In the full sample (*n* = 14,834), approximately half of participants were female, 13% had a mother with a degree, 17% were from the most deprived areas, and 95% were of White ethnicity. However, in the complete-case sample—which includes participants with fully-observed data on all confounders, all exposures and the ‘believes climate is changing’ outcome (*n* = 1,927)—two-thirds of participants were female, 22% had a mother with a degree, 8% were from the most deprived areas, and 96% were of White ethnicity.

Approximately 40% (~6,000) of participants had data on the personality and cognitive ability exposures, although there was little difference between these values comparing the full vs. complete-case samples (Table S4). The means (*M*) and standard deviations (*SD*) for these variables are as follows: extraversion (*M* = 35.3; *SD* = 6.87); agreeableness (*M* = 37.9; *SD* = 5.19); conscientiousness (*M* = 31.9; *SD* = 5.82); emotional stability (*M* = 31.6; *SD* = 6.57); openness to experience (*M* = 35.8; *SD* = 5.65); cognitive ability/IQ scores (*M* = 94.4; *SD* = 13.1).

**Figure 1:**
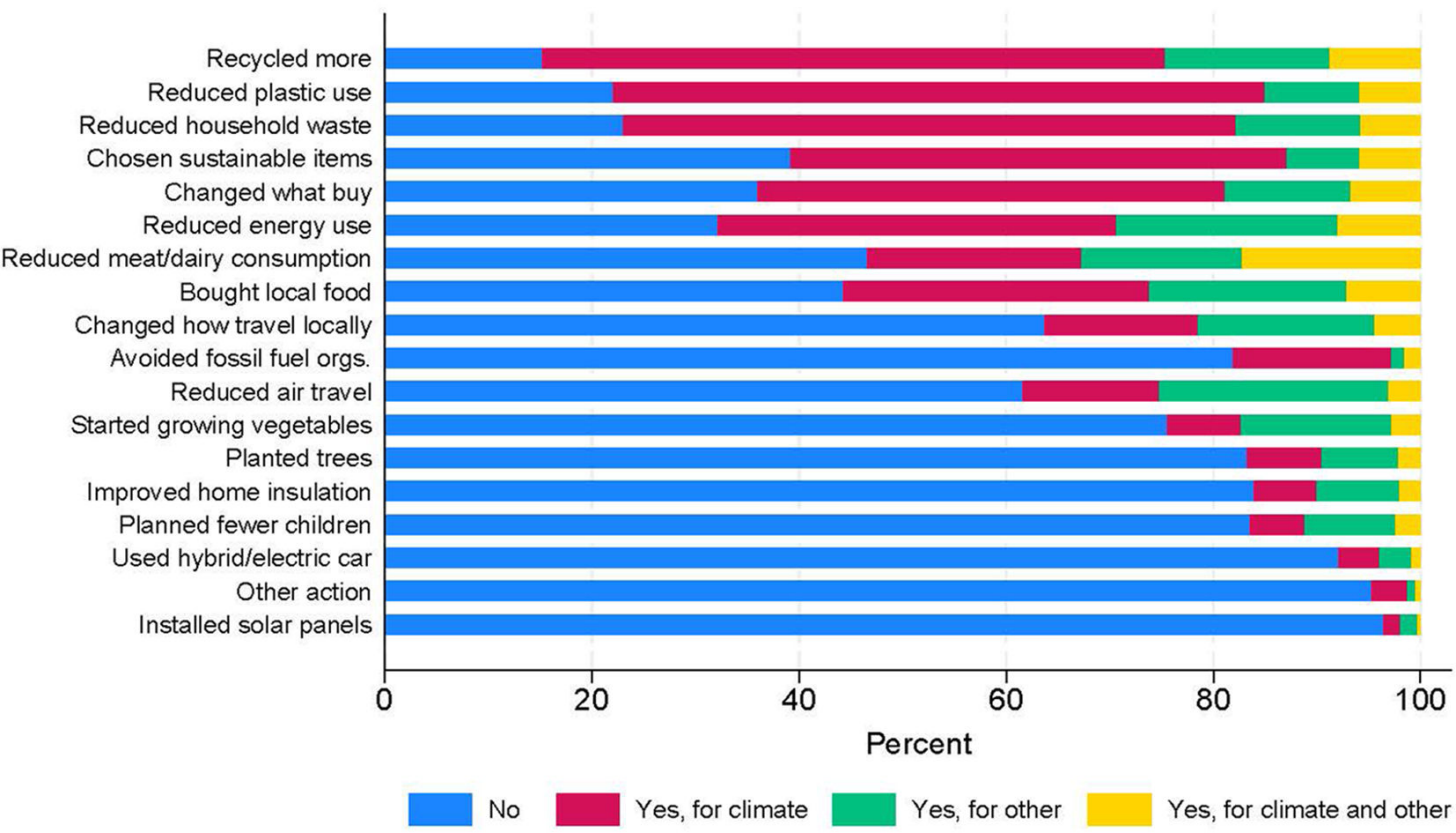
Stacked Bar Chart Showing the Percentage of Individuals Who Engaged in Each of the Pro-Environmental Climate Actions *Note.* (*n* = 2,754 to 4,244). Actions are ranked by the percentage of participants engaging in each action for climate reasons.

Descriptive statistics of the climate beliefs are presented in Table 1, with the climate behaviours summarised in Figure 1 (and Table S5; plus Figure S1 for binary behaviours). Overall, < 2% of this sample believed that the climate was ‘definitely not’ or ‘probably not’ changing, with 90% of individuals ‘somewhat’ or ‘very’ concerned about climate change and < 1% of participants believing that humans were ‘not at all’ to blame for climate change. The actions taken for climate change varied substantially, with more than 60% of participants reducing household waste, recycling/upcycling more and reducing plastic use, to < 20% who changed the way they travelled locally, reduced air travel, purchased/hired electric/hybrid vehicles, improved home insulation, installed solar panels, grew vegetables, planted trees, avoided organisations that support fossil fuel or reduced the number of children planned. On average, participants engaged in 5 of these 17 pro-environmental activities, although a substantial minority of participants (16%) engaged in zero (Figure S2). Approximately 30% (~4,000) of participants had data on these outcomes, and there was little difference in the descriptive statistics of these climate beliefs and behaviours in the full vs. complete-case samples (Tables 1 & S5).

**Table 1:** Descriptive Statistics of the Climate Beliefs Outcomes

Variable	Full sample—*N* (%)	Complete-case sample—*N* (%)
Believes that the climate is changing		
Definitely not	45 (1.1%)	18 (0.9%)
Probably not	44 (1.0%)	15 (0.8%)
Yes, maybe	216 (5.1%)	83 (4.3%)
Yes, probably	545 (12.8%)	232 (12.0%)
Yes, definitely	3,401 (80.0%)	1,579 (81.9%)
*Missing*	10,583 (71.3%)	NA
Concerned about the impact of climate change^a^		
Not at all concerned	97 (2.3%)	44 (2.3%)
Not very concerned	357 (8.5%)	154 (8.1%)
Somewhat concerned	2,028 (48.3%)	906 (47.5%)
Very concerned	1,721 (40.9%)	805 (42.2%)
*Missing*	10,631 (71.7%)	NA
Believes that humans are to blame for climate change^a^		
Not at all	40 (1.0%)	15 (0.8%)
Yes, for some of it	742 (17.7%)	321 (16.9%)
Yes, for most of it	2,076 (49.4%)	913 (47.9%)
Yes, for all of it	1,342 (31.9%)	656 (34.4%)
*Missing*	10,634 (71.7%)	NA
Thinks that personal actions will make a difference to long-term climate changes^a^		
No	879 (20.9%)	405 (21.3%)
Yes	2,193 (52.3%)	1,014 (53.2%)
Not sure	1,125 (26.8%)	487 (25.5%)
*Missing*	10,637 (71.7%)	NA

*Note.* Columns display descriptive statistics for both the full sample (*n* = 14,834) and the complete-case sample with fully-observed data on all confounders, all exposures and the relevant outcome (*n* = 1,905 to 1,927). Note that the percentages of missing data are calculated separately from the observed data.

^a^Individuals who answered ‘definitely not’ to the question “Do you believe that the climate is changing?” did not answer this question.

### Climate Beliefs

**Figure 2:**
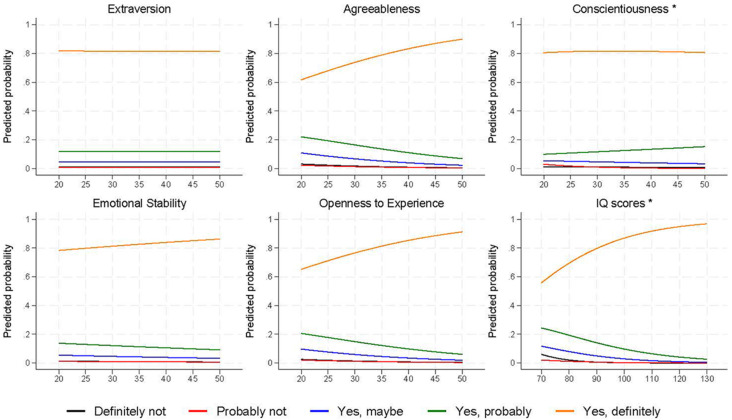
Predicted Probabilities of Answering ‘Believe That the Climate is Changing’ for Each of the Exposure Variables *Note.* Exposure values are on the *x*-axis. Results are based on adjusted ordinal regression models, except where the proportional odds assumption was violated in which case results are from multinomial models (denoted by an asterisk; *n* = 2,327 to 2,426).

In adjusted ordinal models, agreeableness (odds ratio [OR] = 1.07, 95% confidence interval [CI] = 1.04 to 1.09, *p* < .001, pseudo-*R^2^* = 0.9%), openness to experience (OR = 1.06, 95% CI = 1.04 to 1.08, *p* < .001, pseudo-*R^2^* = 1.2%) and IQ scores (OR = 1.06, 95% CI = 1.05 to 1.07, *p* < .001, pseudo-*R^2^* = 5.0%) were all positively associated with belief in climate change (Figure 2). Emotional stability had a weaker positive association (OR = 1.02, 95% CI = 1.00 to 1.04, *p* = .002, pseudo-*R^2^* = 0.2%), with little-to-no association found for extraversion (OR = 1.00, 95% CI = 0.98 to 1.02, *p* = .926, pseudo-*R^2^* = 0.0%) or conscientiousness (OR = 1.00, 95% CI = 0.99 to 1.02, *p* = .666, pseudo-*R^2^* = 0.0%; full results in Table S6). Although the proportional odds assumption was violated for conscientiousness and cognitive ability, multinomial models reported similar results (Table S7).

Comparable patterns of results were found for concern over the impact of climate change (Figure S3), with agreeableness (OR = 1.06, 95% CI = 1.05 to 1.08, *p* < .001, pseudo-*R^2^* = 1.1%), openness (OR = 1.07, 95% CI = 1.05 to 1.09, *p* < .001, pseudo-*R^2^* = 1.7%) and IQ scores (OR = 1.04, 95% CI = 1.03 to 1.05, *p* < .001, pseudo-*R^2^* = 3.0%) strongly associated with greater concern, but weaker and largely null associations for extraversion (OR = 0.99, 95% CI = 0.98 to 1.00, *p* = .136, pseudo-*R^2^* = 0.1%), conscientiousness (OR = 1.00, 95% CI = 0.99 to 1.01, *p* = .992, pseudo-*R^2^* = 0.0%) and emotional stability (OR = 0.99, 95% CI = 0.98 to 1.00, *p* = .197, pseudo-*R^2^* = 0.0%).

This pattern was again repeated for the extent to which humans are to blame for climate change (Figure S4), with agreeableness (OR = 1.03, 95% CI = 1.02 to 1.05, *p* < .001, pseudo-*R^2^* = 0.3%), openness (OR = 1.03, 95% CI = 1.02 to 1.05, *p* < .001, pseudo-*R^2^* = 0.3%) and IQ scores (OR = 1.03, 95% CI = 1.02 to 1.04, *p* < .001, pseudo-*R^2^* = 1.5%) associated with believing humans are to blame, and largely null associations for extraversion (OR = 1.00, 95% CI = 0.99 to 1.01, *p* = .562, pseudo-*R^2^* = 0.0%), conscientiousness (OR = 1.00, 95% CI = 0.98 to 1.01, *p* = .625, pseudo-*R^2^* = 0.0%) and emotional stability (OR = 0.99, 95% CI = 0.98 to 1.01, *p* = .239, pseudo-*R^2^* = 0.0%).

**Figure 3:**
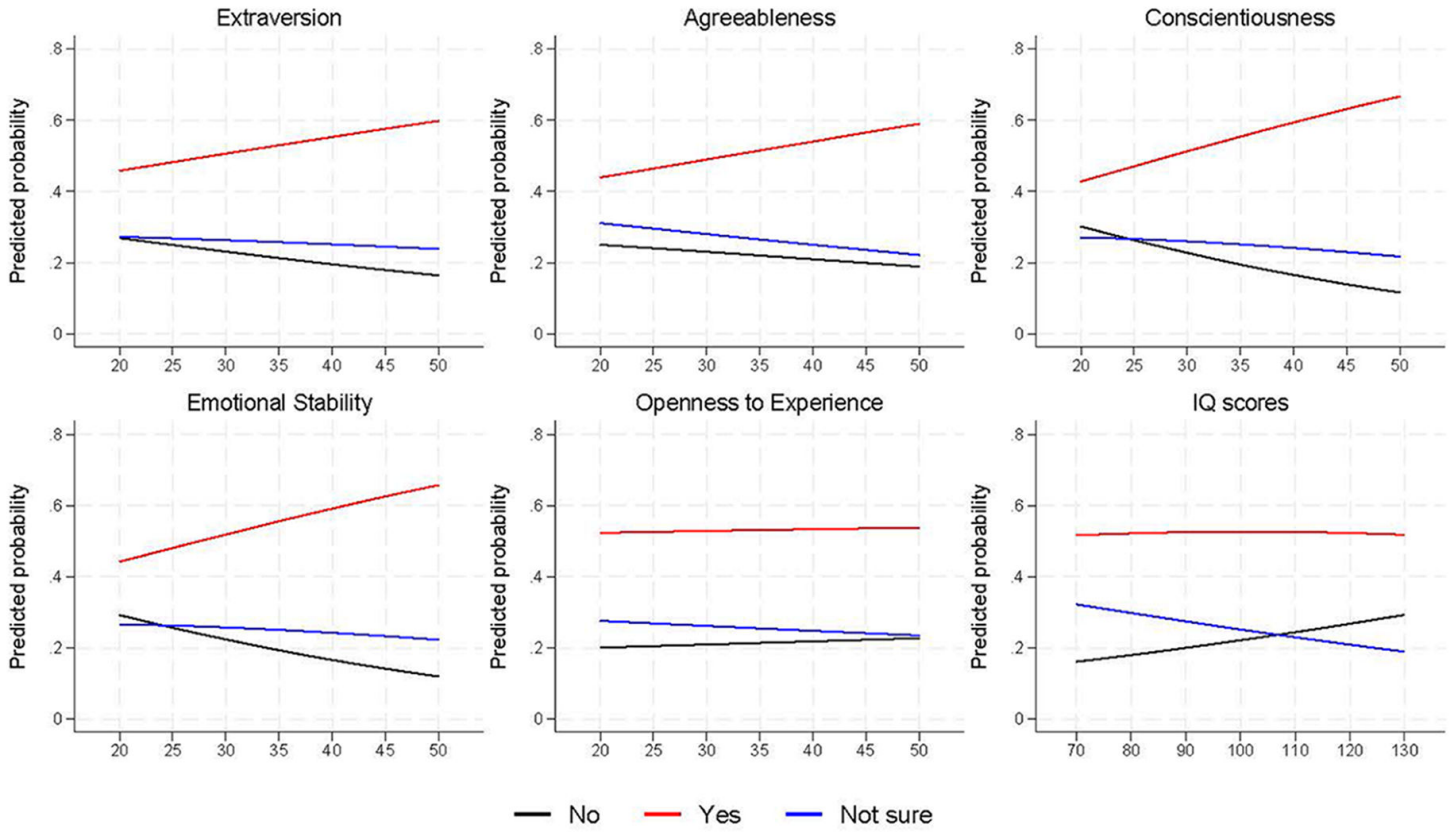
Predicted Probabilities of Answering ‘What You Do, However Small, Will Make a Difference to the Long-Term Effects of Changes to Our Climate’ for Each of the Exposure Variables *Note*. Exposure values are on the *x*-axis. Results are based on adjusted multinomial regression models (*n* = 2,300 to 2,399).

A different pattern of response was found for the question ‘Do you think that what you do, however small, will make a difference to the long-term effects of changes to our climate?’ (Figure 3; Table S8). The strongest associations were found for conscientiousness and emotional stability, with higher scores on these personality traits associated with an increased probability of answering ‘yes’, relative to ‘no’ (extraversion: Relative Risk Ratio [RRR] = 1.05, 95% CI = 1.03 to 1.07, *p* < .001; emotional stability: RRR = 1.05, 95% CI = 1.03 to 1.05, *p* < .001); weaker associations, but in the same direction, were reported for extraversion (RRR = 1.03, 95% CI = 1.01 to 1.04, *p* = .001) and agreeableness (RRR = 1.02, 95% CI = 1.00 to 1.04, *p* = .092). Little association was found for openness (RRR = 1.00, 95% CI = 0.98 to 1.02, *p* = .731), while for cognitive ability, relative to ‘no’ responses, higher IQ scores were associated with a lower probability of answering both ‘yes’ (RRR = 0.99, 95% CI = 0.98 to 1.00, *p* = .025) and ‘not sure’ (RRR = 0.98, 95% CI = 0.97 to 0.99, *p* < .001); from the predicted probabilities from this model (Figure 3), it can be seen there was no association between cognitive ability and ‘yes’ responses, but higher IQ scores were associated with an increased probability of answering ‘no’ and a lower probability of answering ‘not sure’. However, the pseudo-*R^2^* values indicated relatively small improvements in model fit across all these models for this outcome (all < 0.6%).

### Climate Behaviours

**Figure 4:**
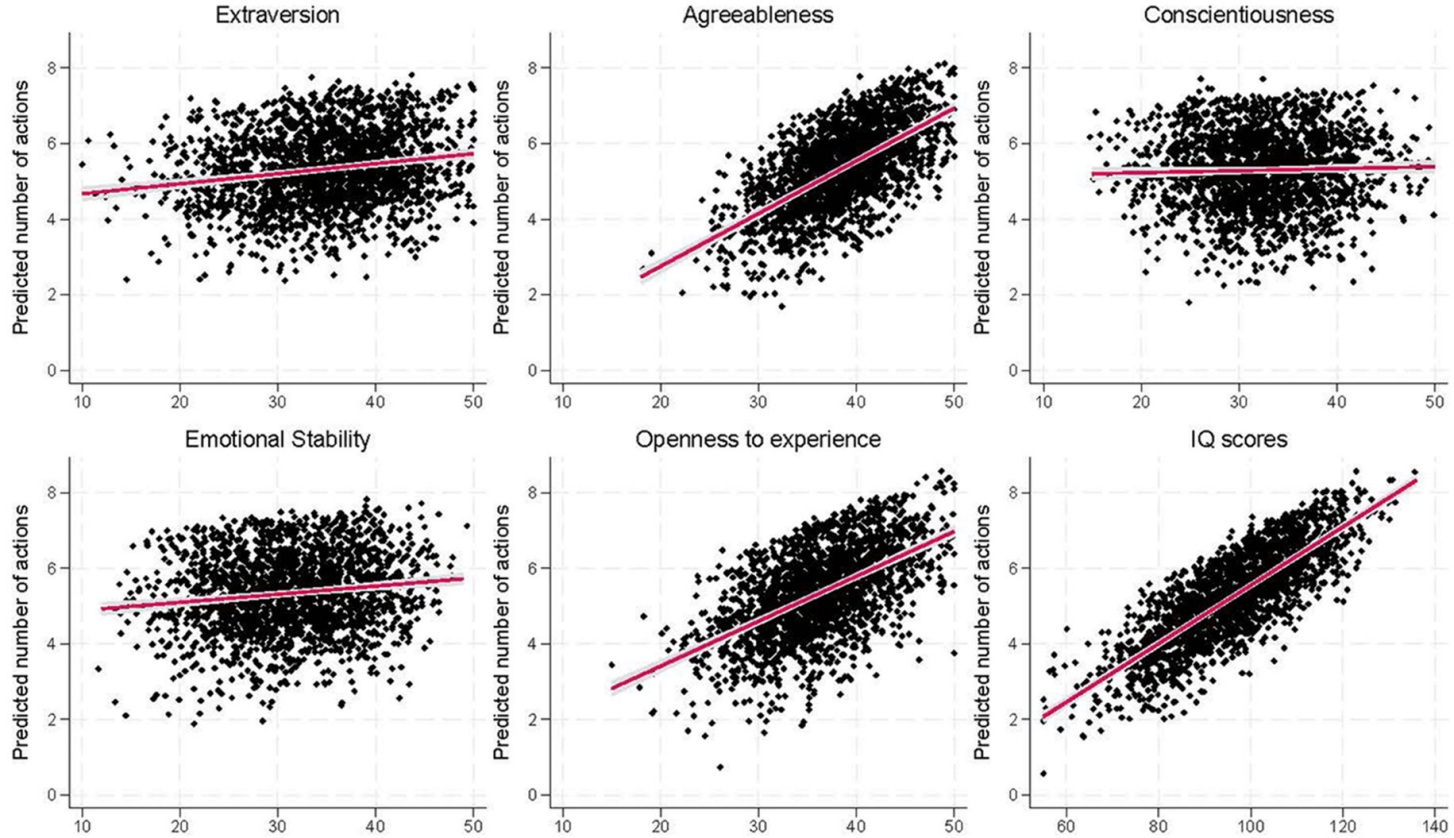
Predicted Number of Pro-Environmental Behaviours Taken Due to Climate Change for Each of the Exposure Variables *Note*. Exposure values are on the *x*-axis. Results are based on adjusted linear regression models. The red line indicates the line of best fit with 95% confidence intervals in grey (*n* = 2,074 to 2,154).

We focus first on the total number of actions that participants report engaging in due to climate change (from 0 to 17). When analysed using a linear model, there was a strong positive association between agreeableness (*b* = 0.11, 95% CI = 0.08 to 0.14, *p* < .001, *R^2^* = 1.8%), openness to experience (*b* = 0.11, 95% CI = 0.08 to 0.14, *p* < .001, *R^2^* = 2.5%) and IQ scores (*b* = 0.06, 95% CI = 0.05 to 0.08, *p* < .001, *R^2^* = 4.1%) and the total number of actions taken (Figure 4; Table S9). Weaker positive associations were reported for extraversion (*b* = 0.02, 95% CI = 0.00 to 0.04, *p* = .043, *R^2^* = 0.2%) and emotional stability (*b* = 0.02, 95% CI = 0.00 to 0.05, *p* = .022, *R^2^* = 0.2%), while there was little-to-no association with conscientiousness (*b* = 0.01, 95% CI = -0.01 to 0.04, *p* = .169, *R^2^* = 0.1%). Results were comparable when using Poisson, negative binomial, zero-inflated Poisson, or zero-inflated negative binomial models (Figures S5–S8; model comparisons in Table S10; full regression results in Tables S11–S14). Results for the actions analysed individually show a similar pattern, with higher agreeableness, openness to experience and IQ scores being positively associated with engaging in many of these behaviours due to climate change (Figures S9–S26; Table S15; with similar findings using binary versions of these pro-environmental behaviours variables; Table S16).

## Discussion

In this paper we have demonstrated robust associations between a number of individual difference variables—namely agreeableness, openness to experience and cognitive ability—and climate-related beliefs and behaviours. We found weaker, and/or more inconsistent, associations with other personality variables, including conscientiousness, extraversion and emotional stability.

These results somewhat support the findings from previous studies—namely the positive association between agreeableness and openness to experience—but failed to replicate positive associations with conscientiousness (Soutter et al., 2020). There are several plausible explanations for this finding. Firstly, conscientiousness appears to have a high degree of geographical heterogeneity—even within US states, there are differences in levels of conscientiousness (Rentfrow, 2010). Furthermore, we examined trait level conscientiousness, rather than individual facets. Previous research has suggested that some of the facets of conscientiousness may work against each other in predicting environmental attitudes and behaviour (e.g., self-discipline and preference for order; Markowitz et al., 2012). Agreeableness may result in greater climate awareness/action as individuals higher in this trait may be more likely to act cooperatively and follow group norms (Wilmot & Ones, 2022). For openness to experience, those who score higher on openness (and specifically the curiosity facet), may be more likely to seek out more sources of information (Jach & Smillie, 2021). Those with higher IQ scores may be more able to critically assess information, and be more resistant to misinformation (Pennycook & Rand, 2020; Ross et al., 2021). Alternatively, the pathway between cognitive ability and environmental beliefs and behaviours could be more indirect. As cognitive ability and educational attainment are tightly linked (Deary et al., 2007), it could be that cognitive ability causes education, and education in turn causes climate knowledge/actions; that is, education may be a mediator on the pathway between intelligence and environmental attitudes and behaviours, rather than cognitive ability causing these beliefs and attitudes directly.

This pattern of results was replicated for the majority of the climate beliefs and behaviours, suggesting that these associations are robust, and that individual differences have similar associations with both environmental attitudes and actions. One interesting exception to this pattern, however, was for the question regarding the impact of one’s individual actions on the impact of climate change. Individuals higher in extraversion, agreeableness, conscientiousness and emotional stability were more likely to believe that their individual actions would have an impact, while openness to experience and IQ scores had no association with agreeing with this statement, and those with higher IQ scores were more likely to *disagree* with it (Figure 3). Perhaps this reflects those with higher IQ scores being able to critically assess climate information, as an individual’s actions are unlikely to have a meaningful impact on climate change; it is only the cumulative impact of large numbers of individuals’ actions which can have such an impact.

To raise awareness of climate change and behaviours to mitigate its impact, this study suggests that targeting and/or designing campaigns to more effectively engage those who score lower on agreeableness, openness to experience and cognitive ability may be effective. For instance, those scoring lower on agreeableness may be less likely to cooperate in the public interest (Wilmot & Ones, 2022); focusing on the self-interested benefits of engaging in pro-environmental actions—such as the health or economic benefits of reducing fossil fuel use, perhaps—may be more effective than campaigns which rely on altruism and self-sacrifice. Similar ideas could also be applied regarding openness to experience and cognitive ability. For instance, as cognitive ability scores are related to processing and understanding information, presenting messages regarding climate change using a range of formats—such as narratives and numerical information—with clear messages may help communicate this information to all (Betsch et al., 2011; Brust-Renck et al., 2013). Similarly, making pro-environmental behaviours seem less ‘radical’ and more consistent with existing behaviours may be more effective for individuals lower in openness to experience. However, although similar interventions based on personality and cognitive ability have been proposed by others (e.g., in relation to health; Deary et al., 2010), these suggestions are very preliminary, rely on these associations being causal, and need to be experimentally tested before being applied in the real-world. Similar approaches have been shown to be effective previously, such as how the effectiveness of climate messaging may vary by cognitive complexity (i.e., thinking about concepts from multiple perspectives; Chen & Unsworth, 2019).

A key strength of this study is the use of large, broadly-representative, longitudinal birth cohort with individual difference exposures measured prior to the climate beliefs and behaviours outcomes. This, combined with the adjustment for a range of plausible baseline sociodemographic confounders, potentially provides stronger evidence for causality compared to previous studies which have tended to be small-scale, cross-sectional, and with minimal adjustment for potential confounders (for several examples of such studies, see Soutter et al., 2020).

However, there are also several limitations. A key limitation is the possibility of unmeasured confounding. While we adjusted for a range of sociodemographic covariates, it is possible that other factors which cause both individual differences and climate beliefs/behaviours may confound these relationships. For instance, educational attainment prior to the individual difference measurements may be such a confounder, as education is associated with higher IQ scores (Ritchie & Tucker-Drob, 2018). Alternatively, factors such as political ideology, which are associated with both individual differences and climate beliefs (at least in predominantly US samples; Drummond & Fischhoff, 2017; Onraet et al., 2015), may also be confounders. However, in both instances the direction of causality between individual differences, climate beliefs/behaviours and the proposed unmeasured confounders (i.e., education or political ideology) is not known with certainty; longitudinal studies which take an explicitly causal approach, which include a wider range of baseline confounders and baseline information on the exposures and outcomes, are necessary to rule out potential unmeasured confounding and provide greater evidence for causality (VanderWeele et al., 2016). Note that measures of educational attainment or political orientation assessed prior to the exposures were not available for this study, so could not be included here as additional confounders.

A further potential limitation is that, although the original ALSPAC sample consisted of ~15,000 offspring, only ~4,000 had data on the climate beliefs and behaviours questions, with only ~2,000 having complete-case data for the analyses. There were also differences in the study characteristics between the full and complete-case samples (Table S3); this missing data and patterns of loss-to-follow-up raises the risk of selection bias potentially biasing our results (Hernán & Robins, 2020; Munafò et al., 2018). While we adjusted for a range of variables known to predict selection in ALSPAC—such as socioeconomic position, offspring sex and ethnicity—which ought to reduce the risk of selection bias, we cannot rule this possibility out.

We also acknowledge the risk of measurement error potentially biasing our results. For instance, due to social desirability bias participants may have over-reported the extent to which they engaged in pro-environmental behaviours, or were less likely to report climate scepticism. This could especially lead to bias if this measurement error was associated with our individual difference exposures (e.g., participants higher in agreeableness or IQ scores being more likely to report socially desirable outcomes). However, some patterns of results did differ by the climate-related question (e.g., the impact of environmental actions, as discussed above), suggesting that answers were unlikely to be solely driven by social desirability. Previous ALSPAC work has also indicated little bias due to social desirability for other potentially-sensitive topics—such as medical history and mental health—when comparing self-reported questionnaires to ‘gold standard’ medical records and clinical interviews (Golding et al., 2001), which together provide some confidence in these results.

Related to the issue of measurement error, there is a large gap between the personality and cognitive ability measures assessed in adolescence (at ages 13 and 15, respectively), and the climate change beliefs and behaviours at approximately age 30. Although previous work suggests that these individual differences are largely stable over the life-course (Atherton et al., 2021; Deary, 2014), they can change over time; for instance, personality may change in response to life events (Bühler et al., 2024) while education can influence IQ scores (Ritchie & Tucker-Drob, 2018). Any such change could perhaps introduce measurement error, potentially weakening any relationships between these exposures and outcomes; for instance, personality traits in adulthood, rather than adolescence, may have a greater impact on climate change beliefs and behaviours. This may be less of an issue for extraversion and openness, which have previously been found to be largely stable from adolescence (age 18) to mid-adulthood (age 40), but may potentially bias associations with conscientiousness, agreeableness and emotional stability, all of which have been found to increase slightly with age (Atherton et al., 2021). While longitudinal data are needed to provide greater evidence for causality, we are somewhat beholden to the ALSPAC data available, which does not include more recent assessments of personality. Nonetheless, we encourage further research using longitudinal data but with a smaller gap between the exposure and outcome to see whether these results replicate.

Finally, as this study was conducted on a sample of young adults from the southwest of England, the extent to which these results are generalisable to a wider population is unknown. For instance, these associations may differ in older generations, in other cities in the UK (Bristol is an especially green city), or in other countries. While similar patterns have been reported across a range of populations (Soutter et al., 2020), additional research is nonetheless necessary to explore how generalisable these results are.

In conclusion, this study has shown that a range of individual differences—namely agreeableness, openness to experience and cognitive ability—are associated with increased awareness of climate change and its impacts, and a greater probability in engaging in a range of pro-environmental behaviours. The extent to which these results are causal, how generalisable they are, and whether they can be used to improve climate literacy, are important open questions for future research.

## Supplementary Materials

For this article, the following Supplementary Materials are available:

- Supplementary tables of study data options and responses. (Freminot et al., 2024)
- Supplementary graphs of descriptive statistics and behavior outcome models. (Freminot et al., 2024)

## Data Availability

Access to ALSPAC data is through a system of managed open access. Information about access to this data is given on the study website (http://www.bristol.ac.uk/alspac/researchers/access/) and in the data management plan (http://www.bristol.ac.uk/alspac/researchers/data-access/documents/alspac-data-management-plan.pdf). Data used for this submission will be made available on request to the Executive (alspac-exec@bristol.ac.uk). The datasets presented in this article are linked to ALSPAC Project Number B4293, please quote this project number during your application. Analysis code and synthetic ALSPAC datasets (created using the ‘synthpop’ R package; (Nowok et al., 2016)), are openly available on DM-S’s GitHub page: https://github.com/djsmith-90/IndivDiffsAndClimate_B2493. As raw ALSPAC data cannot be released, these synthesised datasets are modelled on the original data, thus maintaining variable distributions and relations among variables (albeit not perfectly), while at the same time preserving participant anonymity and confidentiality, thus allowing this research to be ‘quasi-reproducible’ (Major-Smith, Kwong, et al., 2024). Please note that while these synthetic datasets can be used to follow the analysis scripts, as data are simulated they should not be used for research purposes; only the actual, observed, ALSPAC data should be used for formal research and analyses reported in published work.
